# Drying of fecal sludge in 3D laminate enclosures for urban waste management

**DOI:** 10.1016/j.scitotenv.2019.03.487

**Published:** 2019-07-01

**Authors:** Shray Saxena, Babak Ebrazibakhshayesh, Steven K. Dentel, Daniel K. Cha, Paul T. Imhoff

**Affiliations:** Department of Civil and Environmental Engineering, University of Delaware, Newark, DE, United States of America

**Keywords:** Breathable membrane, Fecal sludge, Container-based sanitation, Stagnant film model

## Abstract

Laminated hydrophobic membranes have been proposed as liners for container-based sanitation systems in developing countries. The laminate allows drying of fecal sludge, which might significantly reduce the frequency of container emptying, while containing liquids and solids. While previous laboratory tests demonstrated rapid drying of fecal sludge or water retained in laminates, experiments did not assess the effects of system dimension or scale on performance. In this study fecal sludge drying and water evaporation were evaluated in 3D laminate boxes (decimeter scale) or 3D laminate-lined 40 L and 55 gallon drums (meter scale) that are prototypes of toilet containers for field application. A stagnant film model described fecal sludge drying and water evaporation in the laminate boxes and laminate-lined drums well. The effective diffusion length (*λ*) for the laminate was fitted in all systems and increased with system dimension and scale: *λ* increased by a factor of 1.4 from 1D decimeter-scale envelopes to 3D decimeter-scale boxes, and by a factor of 1.3–1.7 from 3D decimeter-scale boxes to 3D meter-scale drums. The longer *λ* with increasing dimension and scale is likely due to nonuniform temperature and relative humidity in the air outside the laminate and nonuniform temperature within the laminate. Using best-fit *λ* for the laminate-lined 40 L and 55 gallon drum experiments conducted in a controlled laboratory, drying was predicted for an 11-day field experiment. Although the air temperature and relative humidity varied significantly in the field tests from −1 °C to 26 °C and 35% to 97%, respectively, the stagnant film model predicted drying over the 11-day period reasonably well with total error ≤ 13% using 24-h average air temperature and relative humidity. Drying of fecal sludge in laminate-lined drums in the field might be adequately described with a stagnant film model using daily-average weather conditions, if wind speeds are low.

## Highlights

• Fecal sludge (FS) drying examined from 3D laminated membrane enclosures
• Rate of FS drying decreased as scale and dimension of enclosures increased.
• Full-scale laminate-lined toilet containers tested in lab and field tests
• Stagnant film model calibrated to 3D toilet container in lab fitted field data well.

## Graphical abstract

**Figure:**
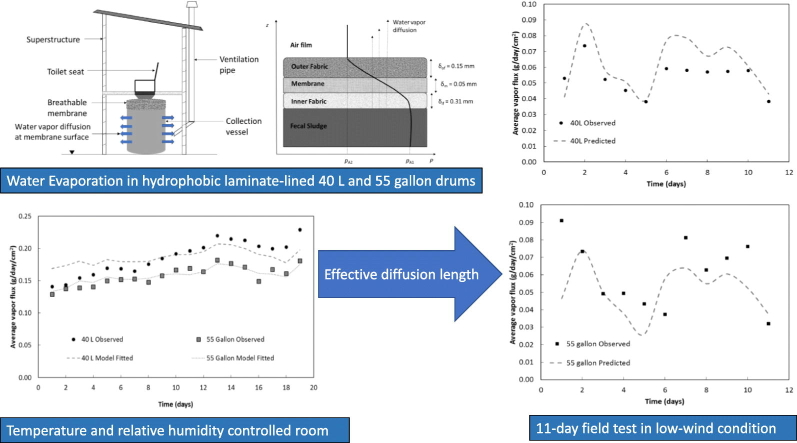

## 1 Introduction

Sanitation systems around the world include conventional sewerage and on-site systems that may include septic tanks, leach pits and pit latrines. Some of these on-site systems were traditionally viewed as temporary solutions pending construction of permanent sewers; however, they now serve 2.7 billion people worldwide as the construction of sewers have not kept pace with rapid urban expansion in low and middle-income countries (Strande et al., 2014). Though these on-site facilities provide a safe and private place for defecation, they have had limited success in many poor urban neighborhoods where narrow, irregular streets cause difficulty in removing fecal sludge using suction trucks (Russel et al., 2015). In such areas, container-based systems, which collect and transport fecal wastes in small sealable containers, have been successfully applied (Tilmans et al., 2015).

In 2015, 2.3 billion people in the world, about 32% of the total global population, did not have access to even basic sanitation services (World Health and Unicef, 2017). A majority of these people were concentrated in regions of Sub-Saharan Africa and Southern Asia where people prefer using wash water and do not require toilet paper. Hence, fecal sludge is defined in this paper to include solid fecal matter, urine, wash water, and some flush water.

Lining container-based toilets with laminated hydrophobic membranes can facilitate drying of fecal sludge at the point of collection (Marzooghi and Dentel, 2014). Laminated hydrophobic membranes typically consist of a membrane made from polytetrafluoroethylene (PTFE), polypropylene (PP), or polyvinylidene fluoride (PVDF), and since these compounds are hydrophobic water and any dissolved ions cannot pass through the membrane. On the other hand, water vapor can readily diffuse across the hydrophobic membrane resulting in increased concentrations of ions and solids inside the membrane enclosures (Zhang, 2006). These membranes have been effectively applied in membrane distillation processes using low-grade waste heat at atmospheric pressure conditions (Marek, 2011). Depending on the application, membranes are sometimes sandwiched between an outer and inner fabric for protection. Examples of such laminated membranes include Gore-Tex™ (W.L. Gore & Associates, Inc., DE, USA) and e-Vent™ (eVent fabrics, Lee's Summit, MO, USA) that are best known for their use in rainwear. Other applications of laminated hydrophobic membranes include use in footwear (Event_Fabrics, 2018) and as a composting cover for organic waste treatment (Goldstein and Satkofsky, 2001).

Marzooghi et al. (2017) recently reported an application of laminated hydrophobic membranes for drying of anaerobically digested biosolids. Under moderate temperature gradients (*∆T* = −2, 2 and 10 °C), the moisture content of biosolids decreased from 97% to 12–30% when 1D drying occurred from decimeter-scale envelopes constructed of GORE Wrap Cover Laminate (W.L. Gore & Associates, Inc., DE, USA). Bakhshayesh et al. (2018) examined repeated 1D drying of fecal sludge from centimeter-scale jars covered with eVent laminate (Laminate P4PS4039-3L, eVent fabrics, Lee's Summit, MO, USA) and found no significant reduction in drying performance over five drying cycles, if a moderate brushing/rinsing cleaning procedure was followed between use. Saxena et al. (2019) proposed a container-based toilet system with the eVent laminate lining containers that allowed drying of fecal sludge during collection, thus reducing the frequency of container replacement and sludge disposal. Applying the stagnant film model with parameters determined from 1D decimeter-scale envelope experiments, Saxena et al. (2019) predicted moisture loss from laminate-lined containers in 10 representative developing countries. Important limitations of these previous studies is that all experiments were small-scale, with laminates on the order of centimeter to decimeter scales; drying occurred under constant, controlled environmental conditions; and designs were only evaluated where drying occurred in one dimension. Thus, the predictions of fecal sludge drying from laminate-lined containers in developing countries by Saxena et al. (2019) have a considerable degree of uncertainty.

The objectives of this study are to evaluate the scale up of breathable laminates to 3D fecal sludge enclosures at intermediate and full scale and their drying performance under changing environmental conditions. The utility of extending a simple stagnant film model to describe drying of fecal sludge from 1D small-scale systems to 3D intermediate and field-scale laminate-lined container setups was also examined. The application of laminated hydrophobic membranes in a container-based sanitation (CBS) toilet system is described first, followed by an overview of the stagnant film model and a description of the experimental methods.

### 1.1 Laminated hydrophobic membranes in container-based sanitation toilet systems

**Fig. 1:**
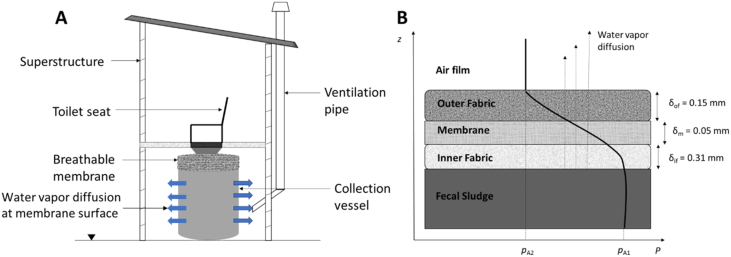
Schematic of the A) proposed container-based system and B) water vapor diffusion across the eVent laminated hydrophobic membrane that lines the container vessel due to a gradient in water vapor pressure (*p*_*A*_).

The proposed toilet, shown in Fig. 1, is a CBS toilet system where a laminated hydrophobic membrane inside the collection vessel contains fecal sludge. The laminate prevents human exposure and leakage to the environment while allowing drying of the fecal sludge by water evaporation through the laminate to the surrounding atmosphere. CBS technologies present several potential advantages over onsite systems, which include easy containment and disposal of fecal matter using sealable containers, and a simplified management strategy for toilet maintenance (Russel et al., 2015). The proposed toilet system is seen as a potential sanitation solution for people with limited space and technology options, such as in informal urban settlements where crowding, extreme poverty, and limited land and infrastructure compel residents to use unimproved and often unhygienic options like pit latrines, hanging toilets, or open defecation (Buttenheim, 2008).

### 1.2 Stagnant film model

Fecal sludge drying in a laminated hydrophobic membrane enclosure is limited by moisture transport through the laminate. Marzooghi et al. (2017) presented a model for moisture transfer through this system, where transport was described separately through the membrane and two protective fabrics in a Gore Wrap Cover Laminate. Although Knudsen diffusion is an important process controlling transport through many hydrophobic membranes (Zhang, 2006), it played a minor role in the Gore Wrap Cover Laminate given the slow rate of molecular diffusion through the thicker protective fabrics (Marzooghi et al., 2017). Instead, mass transfer was adequately described with only molecular diffusion through a single composite layer that accounted for resistance through the membrane and fabrics (Marzooghi et al., 2017). Similarly, Bakhshayesh et al. (2018) determined that the fecal sludge's aqueous phase was blocked by a hydrophobic eVent laminate, while water vapor diffused through the laminated hydrophobic membrane with the flux controlled by gradients of relative humidity and temperature, and the laminate's resistance to diffusion. Bakhshayesh et al. (2018) determined that less than a 2% error in predicted moisture transfer occurred through the three-layer eVent laminate if Knudsen diffusion was neglected, and thus mass transfer was described by molecular diffusion alone (see Supplementary data). Since in this work the eVent laminate is also used, in the analysis below molecular diffusion was assumed to describe moisture transport through laminated hydrophobic membranes.

Water vapor flux across the three-layered laminate (Fig. 1B) was described for the constant rate drying period using a stagnant film model (Sherwood et al., 1975; Marzooghi et al., 2017)

(1) $$N_{A}=\frac{P}{R\left(T_{\mathrm{avg}}+273.15\right)}\frac{D_{\mathrm{AB}}\left(T_{\mathrm{avg}}\right)}{\lambda }\ln\left(\frac{P-p_{A_{1}}}{P-p_{A_{2}}}\right)$$

where *N*_*A*_ is the molar flux of water across the laminate per unit area (mol·m^−2^·s^−1^), *P* is the average total gas pressure across the laminate in Pa, *R* is the ideal gas constant (J·mol^−1^·K^−1^), *T*_*avg*_ is the average temperature across the laminate in °C, *p*_*A*1_ is the water vapor pressure on the fecal sludge side, and *p*_*A*2_ is the vapor pressure on the air side in Pa. The effective diffusion length, *λ* (m), is defined as

(2) λ=δτ/ε

where *δ* is the laminate thickness (m), *τ* is the dimensionless tortuosity, and *ε* is the porosity. The effective diffusion length is a measure of mass transfer resistance through the breathable laminate and was previously fitted to experimental data for 1D drying in centimeter-scale laminate jars (Bakhshayesh et al., 2018) and decimeter-scale envelopes (Saxena et al., 2019). In this study, the effective diffusion length was fitted to data from 3D laminate enclosures at decimeter and meter scales. *D*_*AB*_(*T*_*avg*_) is the diffusivity of water vapor in air (m^2^·s^−1^) and is estimated using the Fuller equation (Gibson, 2000)

(3) $$D_{\mathrm{AB}}\left(T_{\mathrm{avg}}\right)=\left(2.23\times 10^{-5}\right){\left[\left(T_{\mathrm{avg}}+273.15\right)/273.15\right]}^{1.75}$$

At the fecal sludge-laminate interface, the relative humidity is assumed to be approximately 100%. This assumption will be valid as long as free water is present in the sludge (Vaxelaire et al., 2000). Hence, the vapor pressure at the sludge-laminate interface (*p*_*A*1_) is equal to the saturated vapor pressure of water that is estimated with the Arden Buck equation (Buck, 1981)

(4) $$p_{A,\mathrm{sat}}=6.1121\times 100\times \exp\left(\left(18.564-\frac{T}{255.57}\right)\left(\frac{T}{254.4+T}\right)\right)$$

where *T* is the sludge temperature. Vapor pressure at the air-laminate interface (*p*_*A*2_) is also calculated using the Arden Buck equation and the measured relative humidity of air (*RH*)

(5) $$p_{A2}=\left(\mathrm{RH}\%\right)\times p_{A,\mathrm{sat}}$$

## 2 Materials and methods

Though there are many commercially available hydrophobic membranes, in a test of moisture transfer through laminated membranes the highest water vapor flux of 5100 g/day-m^2^ was reported for the eVent laminate, second only to a non-laminated untreated expanded PTFE (ePTFE) membrane (Gibson and Schreuder-Gibson, 2009). Therefore, a three-layered eVent laminate (Laminate P4PS4039-3L, eVent fabrics, Lee's Summit, MO, USA) was selected for this study. This 46.4 μm thick laminate contains a gas permeable ePTFE membrane that is hydrophobic with pore size 0.14 μm determined from BET analysis (Micromeritics ASAP 2020 analyzer). The membrane is supported by a hydrophobic 300D polyester fabric (0.15 mm) on one side and a hydrophilic 20D mesh-like tricot layer backing fabric on the other side. If the hydrophilic fabric of the eVent laminate is placed next to drying sludge, the diffusive pathway for water vapor is smaller than if the hydrophobic fabric contacts the sludge, and drying is enhanced (Bakhshayesh et al., 2018).

The fecal sludge used in this study was collected from student volunteers at the University of Delaware. A 19 L capacity portable toilet with flush capability was used to collect fecal matter. The volunteer collection drive was anonymous and held inside a private unisex toilet for 96 h. The collected fecal sludge included flush-water and urine, but no toilet paper. This sludge was stored in a refrigerator at 4 ± 1 °C for less than a week prior to the start of the drying experiments.

### 2.1 Intermediate scale laminate box experiments

**Fig. 2:**
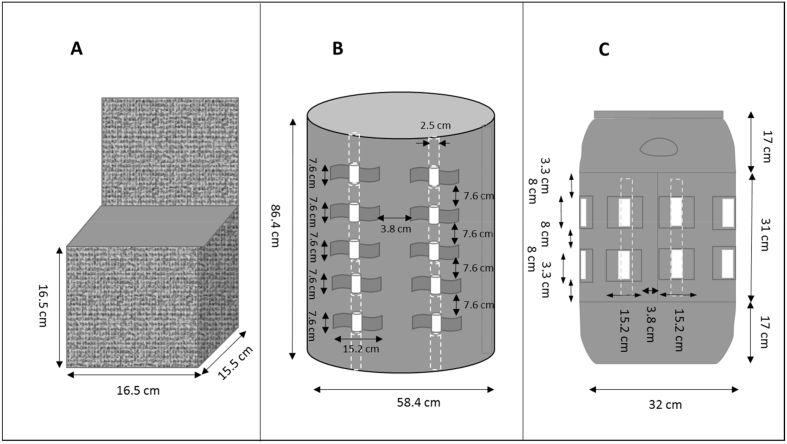
Schematics of laminate enclosures: A) laminate box with laminate lid open, B) 55 gallon drum, and C) 40 L drum. White regions in B and C are exposed PVC pipe that maintains openings also shown in Fig. 3. Not to scale.

Experiments in intermediate scale laminate boxes were conducted to assess fecal sludge drying from 3D enclosures where air flow around each box was unimpeded. These experiments aided our understanding of drying in full scale laminate drums where air flow was expected to be inhibited in some regions. Because the intermediate box experiments were 1.5 L in volume, much less than the full scale laminate drums, experiments using fecal sludge were also possible. Laminate boxes were constructed by shaping a laminated hydrophobic membrane sheet into box structures and sewing the edges with polyester thread (Fig. 2A). To ensure water tightness, the inside and outside edges were covered with silicone glue. Three boxes were designed with a top face cover included in the solid piece of the laminate. This laminate flap was attached to the top of the box using Velcro® on its remaining three edges. The dimensions of each laminate box were 15.5 cm width × 16.5 cm length × 16.5 cm height with a total volume of 1.5 L.

One hypothesis tested in this study is that the stagnant film model adequately describes water vapor transport through laminated hydrophobic membranes lining 3D enclosures at intermediate scale, such as the boxes. While this model described water evaporation and drying of biosolids and fecal sludge when vapor transport occurred in 1D (Sherwood et al., 1975; Marzooghi et al., 2017; Bakhshayesh et al., 2018; Saxena et al., 2019), in 3D enclosures at larger scale nonuniform gradients of temperature and relative humidity inside and outside the enclosure challenge the utility of this modeling approach. The stagnant film model does not account for air flow around the box, nor the buildup of water vapor in the external air along the downwind sides of the box.

To test the hypothesis about the adequacy of the stagnant film model, mass transfer rates were determined for three laminate boxes: two filled with 1 L of fecal sludge (Boxes 1 and 2), and a third control box filled with 1 L of deionized (DI) water. Experiments using water served as a control on the system: by comparing rates of water evaporation with fecal sludge drying, the effect of fecal sludge contaminants on drying was quantified. Experiments with water were also used to estimate the effect of using water versus fecal sludge in the full scale laminate drum experiments described below. Boxes were placed in a constant temperature room sheltered from direct ventilation, although fans continuously mixed room air. The ambient temperature and relative humidity were monitored using MicroDAQ EL-USB-2 *RH*/temp data loggers (Contoocook, NH) with an accuracy of ±0.1 °C and ±2.25%, respectively. The air speed in the vicinity of the boxes was measured with a digital handheld anemometer with a resolution of 0.1 m/s and an accuracy of ±3% (Kestrel 5500 series, WeatherShack, Roanoke, VA, USA). The constant temperature room was kept at 28.9 °C, and relative humidity was maintained at approximately 27% with the assistance of a dehumidifier placed in the room. The boxes were placed 120 cm above the floor and positioned side-by-side on a wire frame shelf approximately 15 cm from each other. The shelf did not impede airflow to the bottom of the laminate boxes.

To mimic the step input of fecal sludge in a field toilet, 1 L of additional fecal sludge was added to Boxes 1 and 2 after 24 and 48 h. For the control box, 1 L of DI water was added at 24 and 48 h. The temperatures of the added fecal sludge and DI water were estimated to be between 21 and 25 °C. Mass loss from each laminate box was measured by periodically weighing the box on a Mettler Toledo XP4002S Balance (Columbus, Ohio) with ±0.01 g accuracy.

Using the measured room temperature and humidity during these drying experiments and assuming the fecal sludge (or water for control) were at room temperature, the stagnant film model (Eq. (1)) was used to determine best-fit *λ* to match the observed drying rates. The ratio of fitted *λ* for fecal sludge drying to water evaporation (*λ*_FS_ / *λ*_DI_) was compared to the previously reported ratio for centimeter-scale tests (Bakhshayesh et al., 2018).

### 2.2 Full scale laminate drum experiments

#### 2.2.1 Container designs

A 55 gallon (208 L) high-density polyethylene (HDPE) drum 59.4 cm ID and 87 cm height was purchased from First State Steel Drum Co (New Castle, DE). These drums are used by the shipping industry worldwide and are readily available in developing countries. The side wall of the drum container was strategically cut (Fig. 2B) to allow natural ventilation around a laminate bag that lined the container. The openings were created by marking rectangular strips around the circumference of the drum at five elevations. The top and bottom of these strips were cut using an electric circular saw. Eight PVC pipes (2.5 cm OD, ~0.76 m length) were fitted into the strips from the top to bottom to create and maintain openings for ventilation. A secondary support plastic mesh (1.27 cm mesh, hardware cloth *Yard Gard*®, Long Grove, IL) was placed between the laminate bag and drum to protect the laminated hydrophobic membrane from the sharp edges of the drum and to enhance ventilation.

A 40 L HDPE drum 36.8 cm ID and 65 cm height was also purchased from First State Steel Drum Co. (New Castle, DE). These smaller drums are also available in developing nations and are easier to manage for disposal purposes when compared to the larger 55 gallon drum. The side wall of this drum was cut similarly as the 55 gallon drum (Fig. 2C). Six PVC pipes (2.5 cm OD, ~30.5 cm length) were fitted into the strips at two elevations to create and maintain openings for ventilation. The amount of open area created by the PVC inserts for the 55 gallon and 40 L drum was about 11% and 8% of the respective drum lateral surface area, respectively. Similar to the 55 gallon drum, the same plastic mesh was used as secondary support and as a spacer between the laminate and the inside of the drum, which permitted ventilation within the container. Because a single piece of laminate was used in the 40 L drum (described below), the plastic mesh was also placed on top of a square frame constructed by joining PVC pipes (2.5 cm OD) to provide a false bottom for supporting the laminate enclosure. A small drain hole (~5.0 cm diameter) was placed at the bottom of each drum to remove pooled water from condensation or leakage.

#### 2.2.2 Laminate design and assembly

The laminate bag for the 55 gallon drum was designed from a single piece of fabric 264.2 cm long x 111.8 cm wide to minimize laminate wastage and reduce the total number of seams (Fig. S1 in Supplementary data). The bag diameter at its base was larger than the diameter of the drum and decreased upwards, so that the downward force applied by fecal sludge did not stretch the sides of the bag as it was filled inside the drum. Once the two seams (seam A and B in Fig. S1) were stitched together, the base was folded into a triangular flap and was sealed facing inward to support the sludge weight at the base. The top of the bag folded over the lip of the drum and contained an extra flap to be supported by clamps if needed. An extension over the extra flap was made into loops for metal bars to be inserted when lifting a full bag. The bag was stitched by Wausau Canvas Company Inc. (Wausau, WI) using small diameter needles and nylon threading with seam tape over the seal holes to ensure waterproofing. The drum lid fit snugly inside the rim of the drum over the folded membrane bag to make the laminate-lined drum a leak-proof system.

For the 40 L drum, a single piece of 1.0 m^2^ laminate was fitted inside the drum and clamped at the drum's mouth, forming an internal bag to collect fecal sludge. By lining the 40 L drum with a single piece of laminate, the cost of sewing and potential leaks from seams in the larger laminate bag fitting the 55 gallon drum were avoided.

#### 2.2.3 Experimental procedures

To study fecal sludge drying in 40 L and 55 gallon drum enclosures, a large volume of fecal sludge would be needed. Due to limited participation from volunteers, collection of such large volumes of fecal sludge was not possible. Hence, to study the constant-rate drying period in meter-scale eVent laminate enclosures, tap water was used to substitute for fecal sludge. Bakhshayesh et al. (2018) showed through centimeter-scale tests that the effective diffusion length of the eVent laminate was approximately 12% larger for fecal sludge drying than water evaporation (λ_FS_ / λ_DI_ = 1.12). Given the relatively small difference in the effective diffusion lengths, water evaporation from the 40 L and 55 gallon drum laminate enclosures was used as a surrogate for fecal sludge drying in these devices. Future field trials in developing countries will use fecal sludge.

**Fig. 3:**
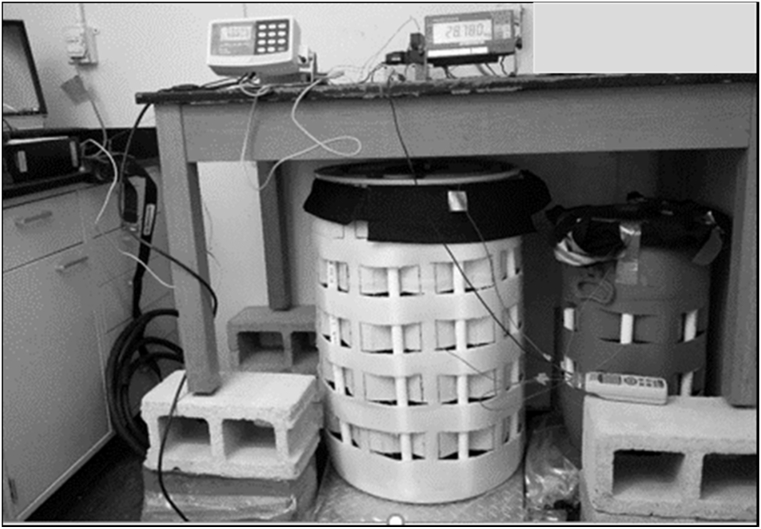
Experimental setup to evaluate drying of water-filled and laminate-lined 55 gallon and 40 L drums in a constant temperature room. The larger 55 gallon drum is shown in the center and the 40 L drum on the right. White PVC pipes that maintain openings for ventilation in each drum are visible. Black eVent laminate is visible at the top of each drum.

Similar to the laminate box tests, drying was measured for water-filled and laminate-lined 55 gallon and 40 L drums placed in the same constant temperature room (Fig. 3). The best fit *λ* was determined from the observed drying rates and compared to 3D decimeter-scale box tests. The 55 gallon drum was placed on a 320D industrial platform scale (±0.05 kg accuracy) from Arlyn Scales (East Rockaway, NY) connected to a data logger (DI 710 Screw Terminal Access DataQ, Akron, Ohio). The 40 L drum was placed on a A&D SB60K11 weighing scale (San Jose, CA) and also connected to a data logger. Ambient temperature and humidity data were recorded by MicroDAQ EL-USB-2 *RH*/temp data loggers (Contoocook, NH) with an accuracy of ±0.1 °C and ±2.25%, respectively, and placed on the top lids of each drum. An Omega RDXL4SD Handheld Thermometer (Norwalk, CT) with ±0.5 °C accuracy and data logger capability was used to record the water temperature (inside laminate) and the air temperature (outer laminate surface) at midpoint height of each drum from thermocouples attached to the laminate surfaces. The constant temperature room was maintained at 30 °C and relative humidity of 25% using a dehumidifier. The air speed in the vicinity of the drums was measured in a similar manner as for the laminate box experiments. Both drums were half-filled with tap water (22.1 °C) on December 23, 2015 and allowed to dry for 45 days.

To evaluate the evaporation rate under more variable field conditions, drying tests with the same laminate-lined 55 gallon and 40 L drums were conducted outside of a building at the University of Delaware (Newark, USA). The drums were placed adjacent to a brick wall and covered with plastic sheeting on the top and sides to prevent direct sunlight and rain, and to reduce the effects of wind (Fig. S2). The drums were filled with tap water (19.4 °C for 40 L and 21.0 °C for 55 gallon drum) on October 8, 2015 and allowed to evaporate for 11 days. Local climatological data for Wilmington, DE were obtained from the National Centers for Environmental Information and compared with the temperature and relative humidity measurements made onsite, which were made in an identical manner as those described above for experiments in the constant temperature room.

While measurements of wind speed were not made during the field experiments, correlations were later developed between the wind speed measured adjacent to the drums using an Adafruit anemometer connected to CR200x data logger (Logan, UT, USA) and wind velocity recorded at the nearest weather station (Newark, DE, USA, elevation: 106 ft; location: 39.6695°N, −75.7503°W). These correlations were used to estimate wind speed adjacent to the drums during the field experiments.

## 3 Results and discussion

### 3.1 Intermediate scale laminate box experiments

**Fig. 4:**
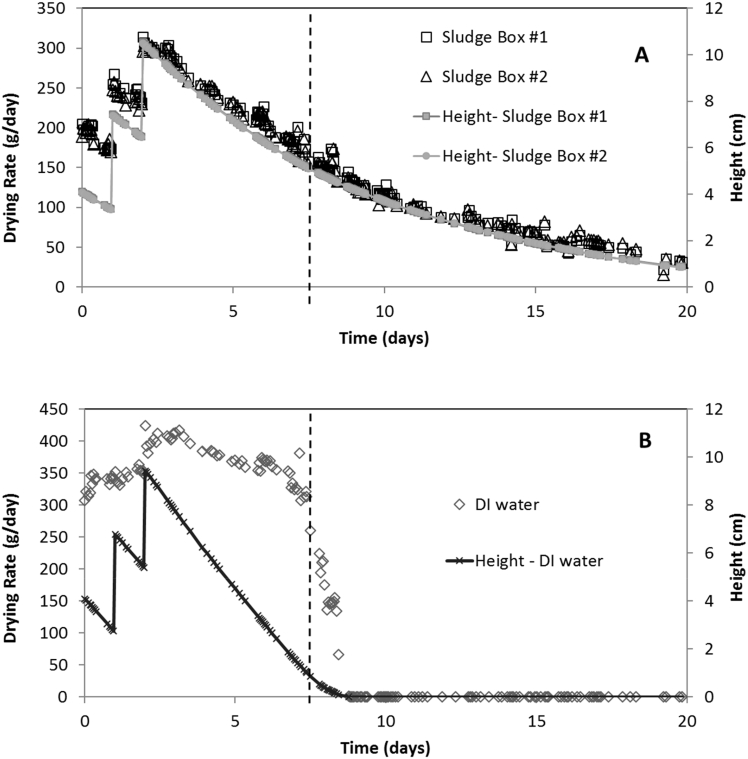
Drying rate and height of liquid in each laminate box for A) fecal sludge and B) DI water (as control) with step-inputs of 1 L at day 0, 1, and 2.

Drying experiments with the laminate boxes were conducted to evaluate fecal sludge drying in a 3D laminate structure. In these boxes, moisture loss from the fecal sludge occurs from the bulk liquid in contact with the laminated membrane as well as vapor transfer from the bulk liquid into the headspace gas phase and then through the laminate lid, which is closed during experiments (Fig. 2A). Fig. 4 presents drying rates of fecal sludge and DI water enclosed in the laminated membrane boxes. The drying rates were calculated from the difference in the observed weights between each measured time. Step addition of 1 L of fecal sludge at 24 and 48 h increased the sludge height, resulting in a 56% and 39% increase in wetted-surface area (*WSA*) inside the container, respectively. The air-gap between sludge level and the top boundary of the laminate box decreased from about 12.4 cm at the start of the experiment to 9.1 cm and 6.0 cm after the first and second step addition, respectively. This air-gap increased as sludge drying progressed after the day 2 step addition, increasing the diffusion length for moisture to escape through the top boundary of the box. For fecal sludge, drying rates peaked at 204 g/day (day 0), 267 g/day (day 1), and finally at 313 g/day (day 2). After each step addition, the moisture loss rate gradually decreased along with the sludge height. The drying rate curve for fecal sludge paralleled the sludge height in the membrane box (Fig. 4A). This result suggests that for fecal sludge drying, the resistance to mass transfer was linearly related to liquid height, which affected both wetted surface area and the diffusion distance through the vapor head space above the liquid level.

While the drying rates of fecal sludge boxes decreased with time, the control box containing DI water showed a relatively constant drying rate until the water depth < ~2 cm. The drying rates in the control box increased slightly after each step addition of water and reached a maximum of 424 g/day after the day 2 step input (Fig. 4B). The water drying rate gradually decreased after the day 2 step addition, just as for fecal sludge experiments, but unlike fecal sludge drying the rate of decrease in drying rate was considerably smaller compared to the rate of decrease in water height. The air speed in the vicinity of the boxes was small and always <0.2 m/s.

It is hypothesized that in experiments with DI water *WSA* was greater than values calculated based on water height alone because of capillarity. Since the inner side of the eVent laminate is a hydrophilic fabric layer, the wicking action of this fabric may cause capillary rise and enhance water evaporation. To test this hypothesis, three initially air-dry laminate strips 4 cm wide and 20 cm long were suspended vertically in a beaker of dyed, DI water. An immediate capillary rise occurred within the first 2 h, which slowed thereafter. The final rise of the dyed water into the laminate after 48 h was 4.4 ± 0.1 cm. A similar experiment with a laminate strip suspended into fecal sludge resulted in a capillary rise of 1.0 cm. The smaller capillary rise in fecal sludge was likely due to the presence of oils and grease that were visible on the liquid surface and likely lowered the contact angle and the air-water interfacial tension. Thus, the reason for the much slower decrease in drying rate with drop in liquid level in water-filled versus fecal sludge-filled laminate boxes is likely due to capillarity. The relative decrease in *WSA* for a given drop in liquid level was smaller for water-filled versus fecal sludge-filled laminate boxes. The effect of capillarity on drying is assessed further below with application of the stagnant film model.

Because the stagnant film model (Eq. (1)) was able to describe biosolids and fecal sludge drying in 1D experiments (Marzooghi et al., 2017; Bakhshayesh et al., 2018; Saxena et al., 2019), we postulate that it may also describe fecal sludge drying in the 3D laminate box experiments during the constant drying rate periods. To evaluate this model, a mass balance equation for the materials retained in the membrane boxes was written in dimensionless form (Marzooghi et al., 2017)

(6) $$\tilde{M}=\frac{\varepsilon }{\tau }\tilde{t}+1$$

where $\tilde{M}$ and $\tilde{t}$ are dimensionless mass inside the laminated membrane enclosure and dimensionless time respectively, and are defined as:

(7) $$\tilde{M}=\frac{M}{M_{i}}$$

(8) $$\tilde{t}=\frac{M_{w}\;\mathrm{WSA}\;P\;D_{\mathrm{AB}}\left(T_{\mathrm{avg}}\right)}{M_{i}\mathrm{\delta R}T_{\mathrm{avg}}}\ln\left(\frac{P-p_{A1}}{P-p_{A2}}\right)t$$

where *M* is the mass of contents inside the laminate enclosure (g), *M*_*i*_ is the mass at the start of the experiment (g), *M*_*w*_ is the molecular weight of water (g/mol), *t* is time (s), and *WSA* is the wetted surface area (m^2^). If the stagnant film model describes fecal sludge drying adequately, then a plot of $\tilde{M}$ versus $\tilde{t}$ should yield a straight line with slope *ε*/*τ* that is identical for all drying periods.

The control box experiment was divided into three time intervals based on step additions: Water1 *t* = 0 to 24 h, Water2 *t* = 24 to 48 h, and Water3 *t* = 48 to 100 h. *WSA* was calculated from the liquid mass and the box dimensions. Marzooghi et al. (2017) reported an inherent −2 °C temperature difference between outside air and biosolids in a laminate enclosure in the absence of a heating element. The lower biosolids temperature was attributed to the cooling effect of liquid evaporation. Though the temperature of the bulk liquid inside the box will vary spatially because of heat transfer through the liquid and across the laminate boundary, the temperature at the liquid/laminate interface (*T*_1_) for all fecal sludge and water drying box experiments was assumed to be 2 °C cooler than the temperature of air adjacent to the outside surface of the laminate (*T*_2_).

**Fig. 5:**
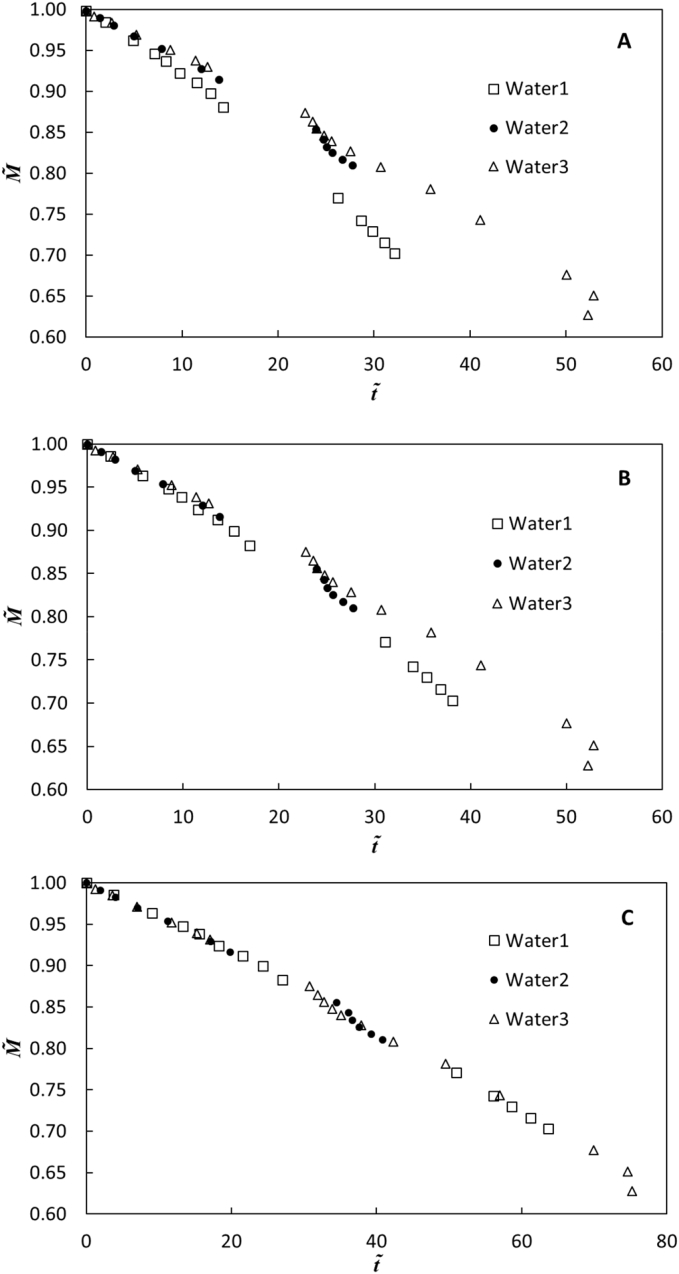
Dimensionless mass ($\tilde{M}$) versus dimensionless time ($\tilde{t})$ of DI water drying in laminate box enclosure assuming A) no capillary rise and *T*_2_ − *T*_1_ = 2 °C for all drying periods; B) no capillary rise, *T*_1_ = *T*_2_ for Water1, and *T*_2_ − *T*_1_ = 2 °C for Water2 and Water3; and C) same temperature conditions as B but capillary rise included. Water1, Water2, and Water3 are drying data after addition of DI water to laminated box on day 0, 1, and 2, respectively. Error bars represent ±SE and are so small as not to be visible.

Using calculated *WSA* and ignoring capillarity, measured $\tilde{M}$ and *T*_2_, and the assumption that *T*_1_ = *T*_2_ − 2 °C, $\tilde{t}$ was calculated from Eq. (8) and $\tilde{M}$ is plotted versus $\tilde{t}$ for DI water drying in the control box in Fig. 5. The three drying periods after the step additions were modeled separately. If the stagnant film model applies, the slope of the plot (*ε*/*τ*) is a fixed property of the laminate and should be identical for all drying periods. However, DI water evaporation for Water1 suggests a faster drying rate when compared to drying periods Water2 and Water3. Water1 cycle was initiated after DI water was allowed to pre-equilibrate in the constant temperature room for 2 h, where temperature = 28.9 °C. Thus, in Fig. 5B *T*_1_ = *T*_2_ was assumed for Water1, since drying might not have proceeded sufficiently long to lower the water temperature in the control box. For Water2 and Water3, though, the addition of fresh and cooler DI water (estimated between 20 and 25 °C) from outside the constant temperature room at 24 and 48 h was believed to help maintain a temperature difference *T*_2_ − *T*_1_ ≈ 2 °C for these drying cycles, and thus this temperature difference was assumed in Fig. 5B. Although this temperature correction improved the data match (constant slope, *ε*/*τ*) between Water1, Water2, and Water3, differences are observed particularly at late time. Finally, the 4.4 cm capillary rise was included in the calculation of *WSA* for all cycles, and these results are shown in Fig. 5C. Here, data from the three cycles collapse to a single line, indicating that both a temperature correction for Water1 and a correction for capillary rise are needed to describe drying with the stagnant film model for these three cycles. Evaporative water flux through the top surface of the box was excluded in these analyses, since the diffusion length through the air space inside the box (above liquid level) was orders of magnitude larger than the diffusion length through the laminate.

Because sludge drying rates in laminate boxes 1 and 2 were nearly identical, averaged data from these two boxes were used at each time interval to calculate the fecal sludge drying rate. With the step-wise input of fecal sludge, drying rate data were divided into three time periods, identical to those for the control box: Sludge1 *t* = 0 to 24 h, Sludge2 *t* = 24 to 48 h, and Sludge3 *t* = 48 to 100 h. Using calculated *WSA* while ignoring capillarity, measured $\tilde{M}$ and *T*_2_, and the assumption that *T*_1_ = *T*_2_ − 2 °C, $\tilde{t}$ was calculated from Eq. (8) and $\tilde{M}$ is plotted versus $\tilde{t}$ for fecal sludge drying in Fig. S3 in Supplementary data. A small difference in slope for Sludge1 compared to Sludge2 and Sludge3 cycles is observed. However, if *T*_1_ = *T*_2_ is assumed only for Sludge1, since like DI water fecal sludge added at day 0 was pre-equilibrated in the constant temperature room, Sludge1, Sludge2, and Sludge3 data collapse to a single line (Fig. S3B in SI). Thus, with an appropriate temperature correction for the first drying cycle, the stagnant film model described fecal sludge drying in 3D laminate boxes. With the addition of a 1 cm capillary rise in the calculation of *WSA* for each cycle, only a minor correction at the end of cycle 3 was observed (Fig. S3). Here, data suggests that temperature correction alone is sufficient to describe drying with stagnant film model for these three cycles.

Based on the adequate fit of Eq. (6) to the laminate box data in Figs. 5C and S3, best-fit *λ* for the eVent laminate were determined using the corrected temperatures for Water1 and Sludge1, and *WSA* that included capillary rise for DI water data (Table 1). The stagnant film model (Eq. (1)) was applied to each drying period, and best-fit *λ* determined by minimizing the sum of squared residuals: *λ*_*FS*_ = 1.16 ± 0.04 × 10^−2^ m and *λ*_*DI*_ = 1.04 ± 0.02 × 10^−2^ m for fecal sludge and DI water, respectively. Here, ± indicates one estimated standard error and were estimated using SolverAid (de Levie, 1999). In comparison, drying in 1D centimeter-scale jars resulted in *λ*_*FS*_ = 4.4 ± 0.2 × 10^−3^ m and *λ*_*DI*_ = 3.94 ± 0.11 × 10^−3^ m for the eVent laminate (Bakhshayesh et al., 2018), while drying in 1D decimeter-scale envelopes constructed from this same laminate resulted in *λ*_*FS*_ = 6.1 ± 0.2 × 10^−3^ m and *λ*_*DI*_ = 5.48 ± 0.13 × 10^−3^ m (Saxena et al., 2019). Across these experiments, best-fit *λ* increased with scale (centimeter-scale jars to decimeter-scale envelopes) and dimension (1D, decimeter-scale envelopes to 3D, decimeter-scale boxes). These increases in the effective diffusion length of 59% and 96% between laminate envelope and box tests for fecal sludge and DI water, respectively, are likely because the assumption of uniform water vapor pressure and temperature on the inside and outside laminate surfaces, as illustrated in Fig. 1B, was violated. In these situations, *λ* becomes a lumped parameter that accounts for diffusional resistance through the laminate and any additional resistance associated with non-uniform temperature and water vapor pressure along laminate inner and outer surfaces While the stagnant film model was able to describe the laminate box data (Figs. 5C and S3), best-fit *λ* for fecal sludge and DI water were larger than that for smaller-scale (jar) or smaller-dimension (envelope) experiments because of the additional resistances associated with the laminate box design.

**Table 1:** Best-fit *λ* for fecal sludge and DI water drying in the laminate boxes. Ambient temperature = 28.8 °C and relative humidity = 28.0%. ± values one estimated standard error (SE). Because of the step-wise input of fecal sludge or water, drying rate data were divided into three time periods for fecal sludge, Sludge1 *t* = 0 to 24 h, Sludge2 *t* = 24 to 48 h, and Sludge3 *t* = 48 to 100 h, and DI water, Water1 *t* = 0 to 24 h, Water2 *t* = 24 to 48 h, and Water3 *t* = 48 to 100 h.

	*λ* (m)
Sludge 1	(1.222 ± 0.003) × 10^−2^ (n = 14)^a^
Sludge 2	(1.08 ± 0.01) × 10^−2^ (n = 13)
Sludge 3	(1.181 ± 0.009) × 10^−2^ (n = 19)
Average	(1.16 ± 0.04) × 10^−2^^b^

Water 1	(1.067 ± 0.006) × 10^−2^ (n = 14)
Water 2	(1.01 ± 0.01) × 10^−2^ (n = 13)
Water 3	(1.036 ± 0.006) × 10^−2^ (n = 19)
Average	(1.04 ± 0.02) × 10^−2^^b^

a n = number of observations.

b The mean *λ* for Sludge and Water are significantly different at 95% confidence level (p = 0.034).

The higher mass transfer resistance for fecal sludge versus DI water (*λ*_*FS*_ / *λ*_*DI*_ = 1.12) in the laminate box experiments is consistent with findings in Bakhshayesh et al. (2018) (*λ*_*FS*_ / *λ*_*DI*_ = 1.12) and is attributed to the presence of solids in fecal sludge. Precipitation and deposition of solids on the laminate as well as sedimentation of particulate matter may alter the path of the escaping vapor flux increasing the effective diffusion length (Marek, 2011). The average vapor flux for laminate boxes was 0.42 ± 0.01 g/day-cm^2^ for DI water but 0.37 ± 0.01 g/day-cm^2^ for fecal sludge (Fig. S4).

### 3.2 Full scale laminate drum experiments

The ratio of open area created by the slits in the drum walls to the initial *WSA* was 13% for both 40 L and 55 gallon drums. Although only 13% of laminate *WSA* was in direct contact with ambient air flow, the air gap between the laminate and the plastic container for the 40 L and 55 gallon drums allowed significant evaporative water flux for experiments in the constant temperature room. In 38 days, 19 kg of water evaporated from the 40 L drum, while over 45 days 62 kg of water evaporated from the 55 gallon drum (Fig. S5). As with the box experiments, the air speed in the vicinity of the drums was small and <0.2 m/s over the duration of the experiments.

**Fig. 6:**
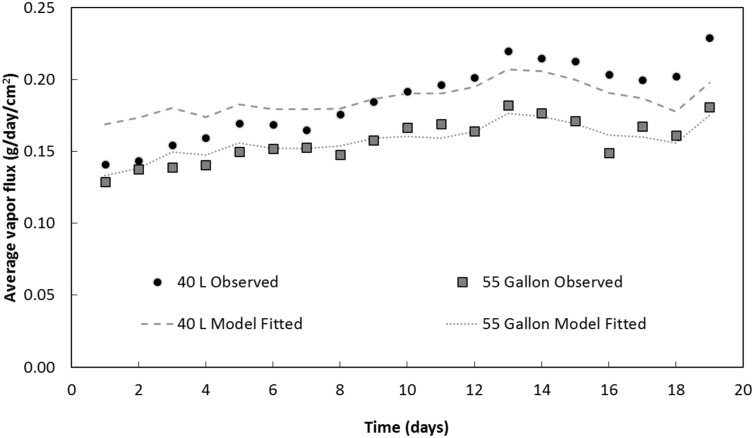
Stagnant film model fitted to observed water vapor flux per *WSA* (g/day/cm^2^) for the laminate-lined 40 L and 55 gallon drums in the constant temperature room.

To evaluate the utility of the stagnant film model for describing these data, air temperature and relative humidity measured by sensors situated on top of each drum and water temperature measured by the thermocouples inside each drum were used. The stagnant film model was only applied to data collected before 20 days (Fig. S6), when water levels remained above the thermocouples. *WSA* was calculated at each time step based on the mass of water in each drum and laminate geometry. The calculated *WSA* included the bottom surface area and the capillary rise of 4.4 cm. Eq. (1) was then fitted to the data by adjusting *λ* alone: best-fit *λ*_*DI*_ = 1.38 ± 0.01 × 10^−2^ m and *λ*_*DI*_ = 1.73 ± 0.01 × 10^−2^ m for the 40 L and 55-gallon drums, respectively (Table 2). The best-fit results of the stagnant film model to evaporative flux data are shown in Fig. 6. The stagnant film model provides a better fit to 55 gallon than 40 L drum data, which may be due to smaller changes in *WSA* for the 55 gallon drum during the experiment: water height decreased 28% (58.2 to 41.6 cm) for the 55 gallon drum, but 54% (24.6 to 11.3 cm) for the 40 L drum over 19 days of drying. The average water vapor flux across the *WSA* was 0.186 ± 0.006 g/cm^2^-day for the 40 L drum, which is 17% higher than 0.158 ± 0.003 g/cm^2^-day for the 55 gallon drum over this period. Both fluxes, though, were still significantly lower than the 0.42 g/cm^2^-day observed for DI water in the membrane box experiment under similar temperature and relative humidity conditions.

**Table 2:** Best-fit *λ* (±SE) for water-filled and laminate-lined 40 L and 55 gallon drums in the constant temperature room.

	*λ* (m)	Ambient temperature (°C)	Water temperature (°C)	Relative humidity (%)
40 L	(1.38 ± 0.01) × 10^−2^ (n = 19)^a^	28.03 ± 0.04	21.1 ± 0.2	25.4 ± 1.4
55 gal	(1.74 ± 0.01) × 10^−2^ (n = 19)	28.45 ± 0.05	21.6 ± 0.1	24.6 ± 1.3

a n = number of observations.

Best-fit *λ* for 55 gallon and 40 L drums are 152% and 216% larger than best-fit *λ* determined for water evaporation in 1D decimeter-scale envelope tests (Saxena et al., 2019), and 33% and 67% larger than *λ* fitted to the 3D decimeter-scale control laminate box. Similar to the transition from 1D centimeter-scale jar experiments to 1D decimeter-scale envelope experiments where best-fit *λ* increased by a factor of 1.4, the transition from 3D, decimeter-scale box experiments to 3D, meter-scale drum experiments resulted in best-fit *λ* increasing by a factor of 1.3–1.7. The longer effective diffusion lengths for the drum experiments may be due to dead zones of air between drum walls and the outer laminate surface, where flow of air was impeded and well-mixed conditions on the outer laminate surface (Fig. 1B) could not be maintained. Air temperatures measured in this dead zone in both drums were 5 to 7 °C lower than room air temperature used in the stagnant film model (Fig. S6), indicating that well-mixed conditions were not maintained.

Drum drying experiments were also conducted outside to test the stagnant film model under field conditions using *λ* obtained from the constant temperature room experiments. The wind speed for the outside field tests varied over the 11-day test, but was small with mean wind speed < 0.2 m/s, similar to what was found inside the constant temperature room. The plastic sheeting that provided a roof and side enclosure limited wind speed in the field test. Similar conditions may occur for some laminate-lined toilets in urban areas. The drum water temperatures, the measured air temperature and relative humidity, and temperatures and relative humidity from weather station data over the 11-day drying experiments are presented in Figs. S7 and S8. Both weather station data and the measured water and air temperatures exhibited similar diurnal patterns. Since local climatological data is easily accessible for most field locations, weather station temperature and relative humidity data were used for field testing of the predictive model. The daily average temperatures of water inside the drums were used in the stagnant film model. In these field tests, the daily mean water temperature differed from the daily mean air temperature, sometimes higher sometimes lower, by an average of 0.8 ± 0.3 °C and 1.0 ± 0.4 °C for the 40 L and 55 gallon drums, respectively.

**Fig. 7:**
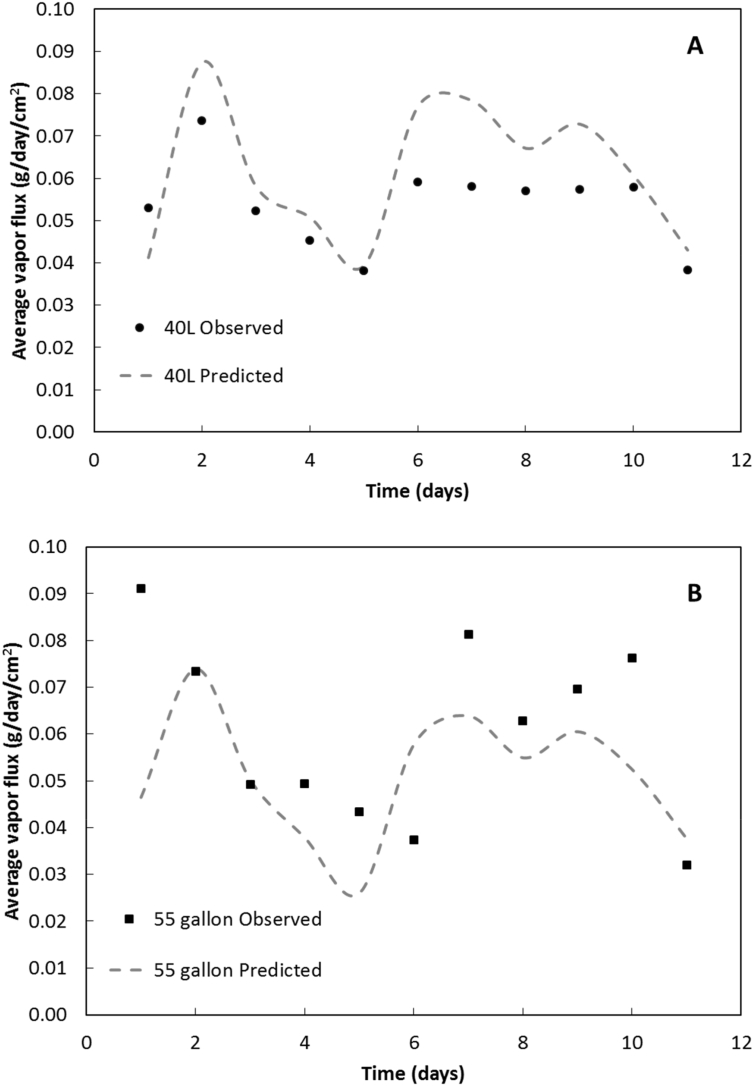
Observed and predicted daily average water vapor flux (g/day/cm^2^) for a laminate-lined A) 40 L drum and B) 55 gallon drum in the 11-day field test.

Daily average evaporative flux rates for the field experiments are shown for the 40 L and 55 gallon drums in Fig. 7. The evaporative water flux varied by a factor of 2 and 3 for the 40 L and 55 gallon drums, respectively, and is associated with changes in temperature and relative humidity. Over the 11 day period, the air temperature and relative humidity ranged from −1 °C to 26 °C and 35% to 97%, respectively, while water temperatures ranged from 4.8 °C to 20.4 °C for the 40 L and, 5.8 °C to 21.1 °C for the 55 gallon drum (see Figs. S7 and S8). Daily average drying rates per *WSA* (including the capillary fringe) were 0.054 ± 0.003 g/day-cm^2^ and 0.061 ± 0.006 g/day-cm^2^ for 40 L and 55 gallon drums, respectively. These drying rates for 40 L and 55 gallon drums are 71% and 61% smaller, respectively, than similar drying rates measured in the constant temperature room and are attributed to the 54% lower mean temperature (13.1 ± 0.1 °C) and 188% higher mean relative humidity (69.4 ± 0.2%) for the 11-day field test.

Similar to the drum tests in the constant temperature room, the stagnant film model was applied to experiments conducted outside using weather station data for temperature and relative humidity, the measured water temperatures, and best-fit *λ* reported above from the drum experiments in the constant temperature room. Model-predicted evaporative water fluxes for the 40 L and 55 gallon drums are shown in Fig. 7A and B, respectively. Over 11 days of the outside drying experiments, if temperatures and relative humidity are averaged over each 24-h period and mean values used in the stagnant film model, the mean absolute error in predicting daily-average vapor flux is 17 ± 3% and 24 ± 5% for the 40 L and 55 gallon drums, respectively. If instead total drying is predicted for the entire 11 days using 24-h average measurements of temperatures and relative humidity, the model-predicted error in drying rate reduces to −13% and 11% for the 40 L and 55 gallon drums, respectively. Despite the significant variability in weather conditions, the stagnant film model predicted drying over the 11-day period reasonable well with total error ≤ 13% using 24-h average parameters. This suggests that drying of fecal sludge in similar laminate-lined drums in the field might be described adequately using daily-average weather conditions and sludge temperatures.

## 4 Conclusions

Experiments were conducted to evaluate the utility of laminated hydrophobic membranes for retaining and allowing moisture transfer from fecal sludge and water in laminate-lined containers. Building upon previous studies of fecal sludge and water drying in 1D centimeter-scale jar and 1D decimeter-scale envelope experiments, drying tests were conducted in 3D decimeter-scale laminate boxes and 3D meter-scale laminate-lined drums that are prototypes for field deployed laminate-lined toilet containers. The stagnant film model was used to describe drying in these experiments, where *λ* is an effective diffusion length for water evaporation through the laminated membrane. Similar to the transition from 1D centimeter-scale jar experiments to 1D decimeter-scale envelope experiments where best-fit *λ* increased by a factor of 1.4, the transition from 3D decimeter-scale box experiments to 3D meter-scale drum experiments resulted in best-fit *λ* increasing by a factor of 1.3–1.7. The longer effective diffusion length with increasing experimental scale is likely due to nonuniform temperature in the fecal sludge or water retained in the laminate enclosure and similar nonuniform temperature and relative humidity in the air surrounding the laminates. Fecal sludge drying in the decimeter-scale boxes occurred more slowly than moisture loss from water-filled boxes, with best-fit *λ*_*FS*_ / *λ*_*DI*_ = 1.11. The slower drying rate from fecal sludge is attributed to precipitation and deposition of solids onto the laminate as well as sedimentation of particulate matter, which may alter the path of the escaping vapor flux and increase the effective diffusion length (Bakhshayesh et al., 2018).

Best-fit *λ* determined for water evaporation from the prototypes for laminate-lined toilet containers (40 L and 55 gallon drums) in a constant temperature room were used to predict drying in identical drums placed outside over 11 days. While outside temperature and relative humidity varied by a factor of 24 and 3, respectively, over the 11 days, the stagnant film model using laboratory-determined *λ* and daily average temperature and relative humidity described field drying rates well with overall error ≤ 13%. The stagnant film model does not account for wind, which may alter the effective diffusion length for water vapor transport in the vicinity of the laminate. Under higher wind speeds, for example, turbulence in the vicinity of the drums is enhanced and mixing in the air gap between the laminate and plastic wall of each drum would increase, which would cause *λ* to decrease. Over the 11-day field test, wind speeds in the vicinity of the drums were <0.2 m/s because of the position of the drums with respect to an adjacent brick wall and plastic sheeting that formed a roof and side walls. For the low wind speeds during this field test, the stagnant film model that neglects wind successfully predicted drying in the laminate-lined drums. It was necessary, however, to use the system-dependent *λ* determined from prototype tests in the laboratory. These results suggests that fecal sludge drying from laminate-lined toilets might be predicted with a reasonable degree of accuracy using the simple stagnant film model with readily available atmospheric parameters, i.e., air temperature and relative humidity, if wind speeds are low in the vicinity of the laminate-lined toilet container. The only limitation is that the effective diffusion length for water evaporation, *λ*, must be determined independently for each laminate-lined toilet design.

Based on the experiments reported herein, over an 11-day drying period the laminate-lined prototype toilets lost 9 and 7% of their mass from water evaporation, for the 40 L and 55 gallon drums respectively. The drying rate achieved in field applications will be a function of the local climatic conditions, i.e., air temperature and relative humidity, and could be considerably higher in warmer and more arid climates.

In field applications, the temperature of the fecal sludge will not be known, as was the case in the field experiments reported here for water evaporation. The temperature of fecal sludge in most cases is expected to be higher than the air temperature, since fresh fecal sludge and urine is near body temperature (37 °C) and biological activity within fecal sludge generates additional heat when retained in toilets. Thus, assuming the temperature of the fecal sludge is identical to air temperature would result in a conservative (low) estimation of drying rate. For the field experiments reported in this work, if the water temperature inside the drums was assumed equal to the air temperature the overall error in drying rate would increase from ≤13% to ≤23%. A more robust model that accounts for heat generation within fecal sludge, heat transfer, and wind effects around the toilets might improve drying-rate predictions for container-based systems employing laminated hydrophobic membranes, particularly in locations where toilets are well-ventilated. Finally, field testing of fecal sludge drying from laminate-lined toilets is needed to confirm the utility of the stagnant film model and to explore other issues that may affect usage, e.g. emission of odorants.

## Appendix A Supplementary data

Supplementary material Image 1
