# Supplementary material for: Drying of fecal sludge in 3D laminate enclosures for urban waste management
Source: Sci Total Environ. 2019 Jul 1;672:927–37. doi: 10.1016/j.scitotenv.2019.03.487 (PMC6525274; doi:10.1016/j.scitotenv.2019.03.487)
Supplement: Supplementary file 1 — Supplementary material [file mmc1.docx]

**Supplemental Data for**

**Drying of Fecal Sludge in 3D Laminate Enclosures for Urban Waste Management**

Shray Saxena^1^, Babak Ebrazibakhshayesh^1^, Steven K. Dentel^1^, Daniel K. Cha^1^, Paul T. Imhoff^1*^

^1^Department of Civil and Environmental Engineering, University of Delaware, Newark, DE

^*^Corresponding Author – Email: imhoff@udel.edu

1. **Importance of Knudsen diffusion in laminated hydrophobic membrane**

To estimate the importance of Knudsen diffusion for water vapor transport through the eVent™ laminate (Laminate P4PS4039-3L, eVent fabrics, Lee's Summit, MO, USA), transport through air films in the three-layer laminate was considered. Contact angle tests confirmed that the membrane and outer fabric of the eVent Laminate were hydrophobic and thus should remain air-filled. However, the inner fabric in contact with fecal sludge or DI water was hydrophilic and was expected to fill with liquid during the drying experiments.

The procedure for estimating the importance of Knudsen diffusion was as follows. The conceptual model for water vapor transport through the laminated hydrophobic membrane described in equations (1-2) of Marzooghi *et al.* (2017) were assumed to apply. Resistance to moisture transfer through the inner fabric was assumed negligible, since fabric openings were filled with liquid during experiments. The thickness of the outer fabric in contact with air, $\delta_{of}$ and the membrane thickness, $\delta_{m}$, were measured and are reported in Table S1. The resistance coefficients, $C_{i},$for gas-phase diffusive transport in each component $i$of the laminate (outer fabric, and membrane) in equation (2) of Marzooghi *et al.* (2017) were estimated from an expression derived for diffusive water vapor transport through direct contact membrane distillation (Khayet et al., 2004).

$C_{i}=\frac{1}{RT_{avg}\delta_{i}}\left[ \frac{3\tau_{i}}{2\varepsilon_{i}\left\langle r_{i} \right\rangle}\left( \frac{\pi M_{A}}{8RT_{avg}} \right)^{0.5}+\frac{P_{B}\tau_{i}}{\varepsilon_{i}PD_{AB}} \right]^{-1}$ (S1)

where *R* is the ideal gas constant; $T_{avg}$ is average temperature in the laminated membrane; $\tau_{i}$, $\varepsilon_{i},$ and $\left\langle r_{i} \right\rangle$ are the tortuosity, porosity, and mean pore radius for laminate component $i; M_{A}$ is the molecular weight of water; $P_{B}$ is the pressure of the air in the gas phase that consists of air and water vapor; $P$ is the total gas phase pressure; and $D_{AB}$ is the molecular diffusion coefficient of component *A* (water vapor) in component *B* (air) and was estimated with the Fuller equation $D_{AB}=(2.23\times10^{-5})\left[\left(T_{avg}+273.15\right)/273.15\right]^{1.75}$ (Gibson, 2000). The first term in the bracket in equation (S1) describes the effect of Knudsen diffusion while the second term molecular diffusion.

The mean pore radius, porosity and tortuosity for the membrane were measured and are reported in Table S1. Mean pore radii for the outer fabric were estimated from SEM images to be within 2$5<\left\langle r_{of} \right\rangle<50$ µm. Porosity of the outer fabric was unknown, so a range was used in the calculations: 0.3 < $\varepsilon_{of}<0.9$. The tortuosity for the outer fabric was assumed as 5. These parameter values were used in equation (S1) for the experimental conditions of the tests ($P=101$ kPa and $T_{avg}varied between 5-35$^o^C) to estimate $C_{m}$and $C_{of}$.

The relative error neglecting Knudsen diffusion was estimated with

$\text{relative error}= \left| C_{l}^{k+m}-C_{l}^{m} \right|/C_{l}^{k+m}$ (S2)

where $C_{l}^{k+m}$ is the resistance coefficient for the laminate determined from equation (2) of Marzooghi *et al.*, (2017) including both Knudsen and molecular diffusion for $C_{m}$and $C_{of}$, while $C_{l}^{m}$is the same coefficient computed assuming only molecular diffusion occurs through the membrane and fabric. No resistance was assumed for the inner fabric in equation (2) of Marzooghi *et al.*, (2017), since inner fabric pores were filled with liquid. Over the range of parameters investigated, the relative error never exceeded 2% when neglecting Knudsen diffusion through the laminate. Thus, for the experimental conditions in this study and when fluid pressures are not sufficient to penetrate the membrane, molecular diffusion dominates the transport of water vapor and Knudsen diffusion can be neglected.

Table S1. Properties of fabrics and membrane in eVent™ P4PS4039-3L Laminate.

| Material | Membrane or Fabric  Thickness^1^ | Pore Radius | Porosity | Tortuosity |
| --- | --- | --- | --- | --- |
|  | (mm) | (µm) | (-) | (-) |
| Membrane | 0.046 | 0.14^2^ | 0.84 | 1.6 |
| Outer fabric | 0.15 | 25 – 50^3^ | 0.3 – 0.9^4^ | 5^4^ |

^1^ Measured with micrometer

^2^ Pore size estimated from nitrogen adsorption at 77 K using the Micrometrics ASAP 2020 (Micrometrics Instruments, Co., Georgia, USA) and the BJH method.

^3^ Estimated range from SEM images of fabric.

^4^Assumed range or value.

1. **Membrane Design**


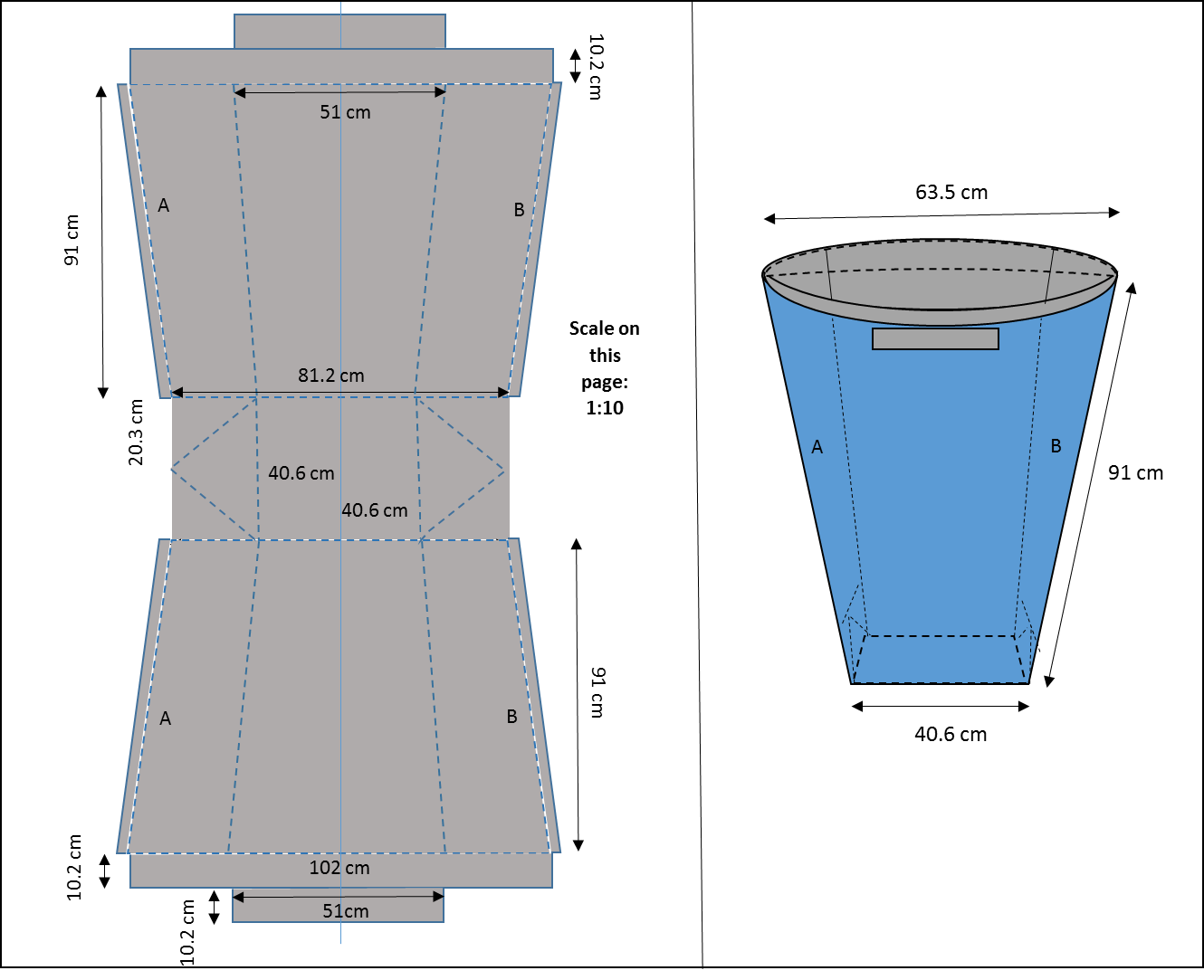


Figure S1. Design for sewing of laminated hydrophobic membrane for the 55-gallon drum. To make the laminate bag (right figure), Side A-A and B-B (left figure) are stitched first, then the base is folded along the triangular vertices and stitched to the adjacent inside face of the bag.

1. **Full-Scale Membrane Drum Outside Test - Setup**


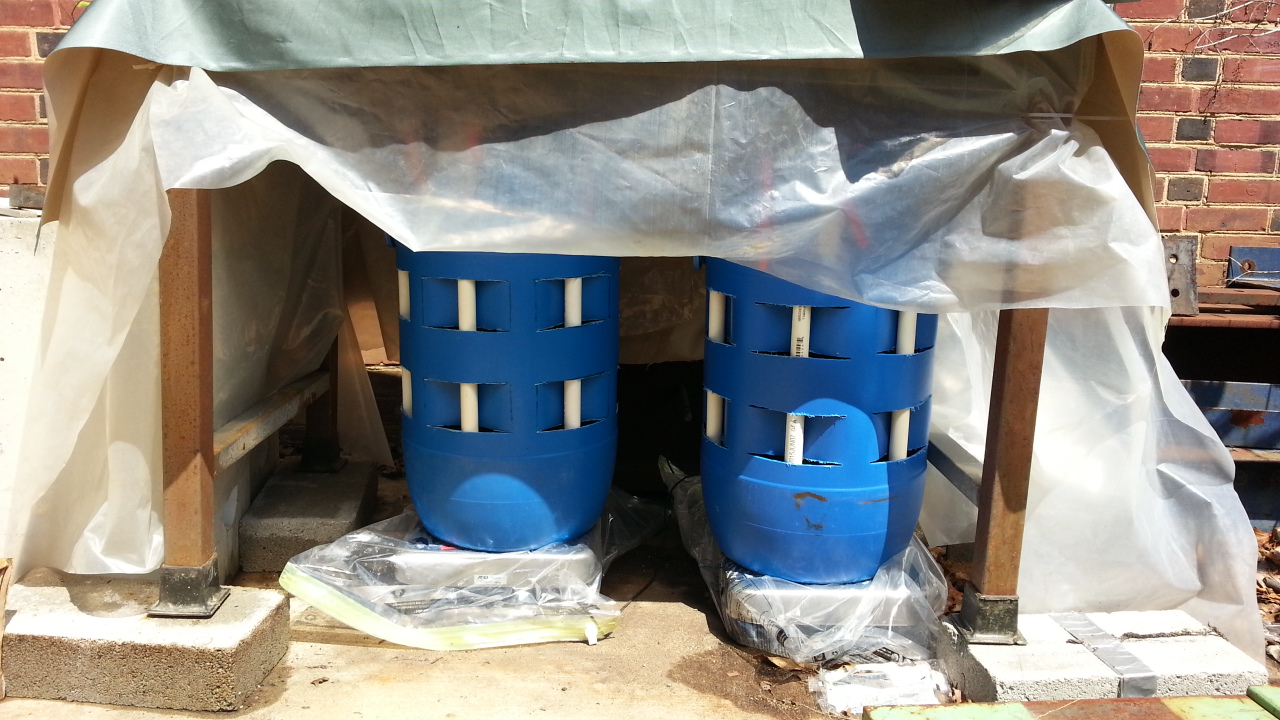


Figure S2. Field experimental setup for two laminate-lined 40 L drums conducted at University of Delaware (Newark, USA). The two drums were placed on weighing scales adjacent to a brick wall and covered from the top and sides using a plastic sheet. Each weighing scale was placed inside a plastic bag and then calibrated using standardized weights before the experiment. Temperature probes were attached to each laminate surface through the slits in the drum wall. The computer and data logger used to record changes in drum weight was kept inside the building and connected to the weighing scales through a window near the experimental setup. The 55 gallon drum replaced the left-side 40 L drum for the field test experiment.

1. **Intermediate-Scale Laminate Box Experiment**


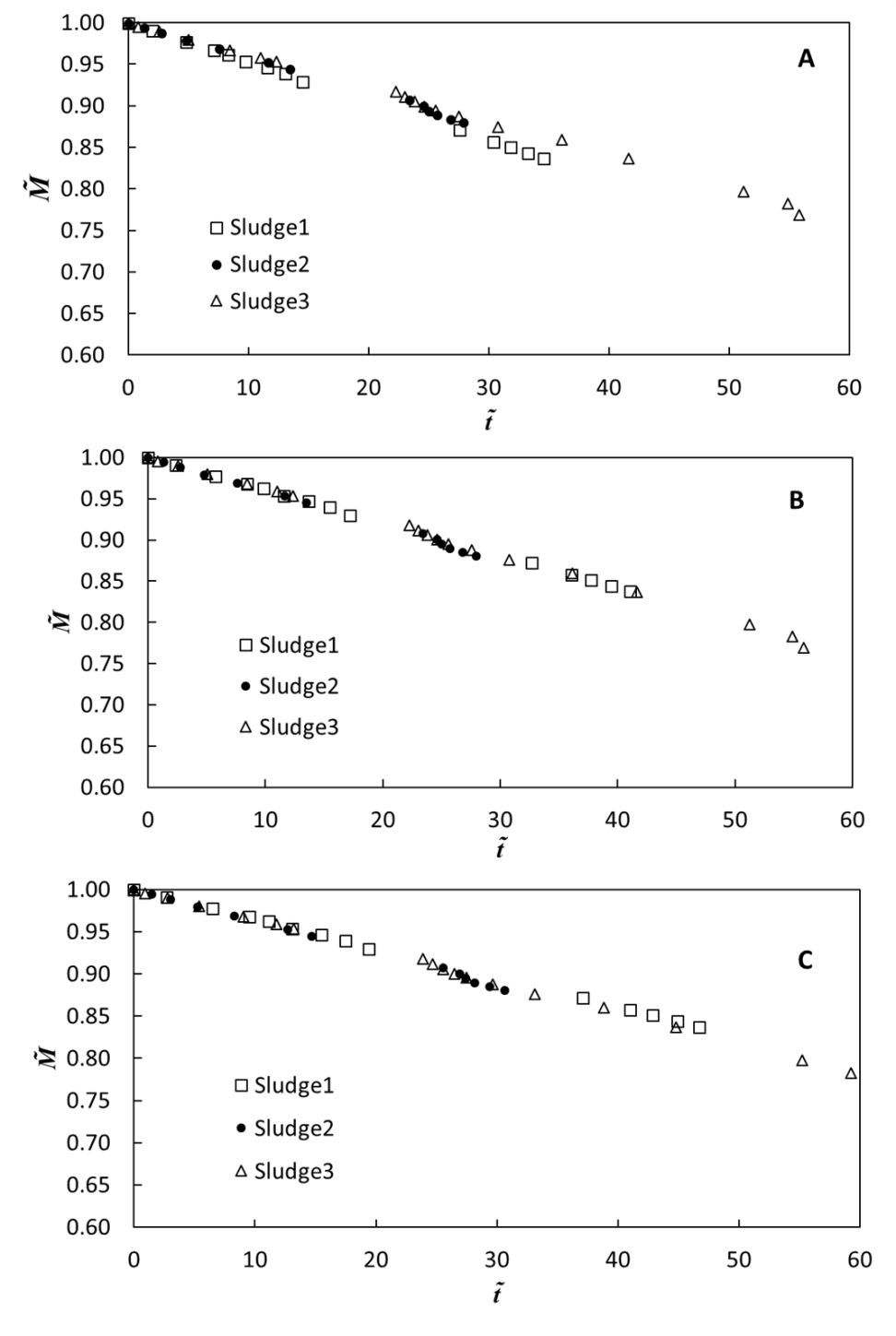


Figure S3. Dimensionless mass ($\tilde{M}$) versus dimensionless time ($\tilde{t})$ of fecal sludge drying in a laminate box enclosure with A) *T_2_* – *T_1_* = 2 °C for all drying periods; B) *T_1_* = *T_2_* for Sludge1, and *T_2_* – *T_1_* = 2 °C for Sludge2 and Sludge3; and C) same temperature conditions as B but capillary rise included. Sludge1, Sludge2, and Sludge3 are drying data after addition of fecal sludge to laminated boxes on day 0, 1, and 2, respectively.


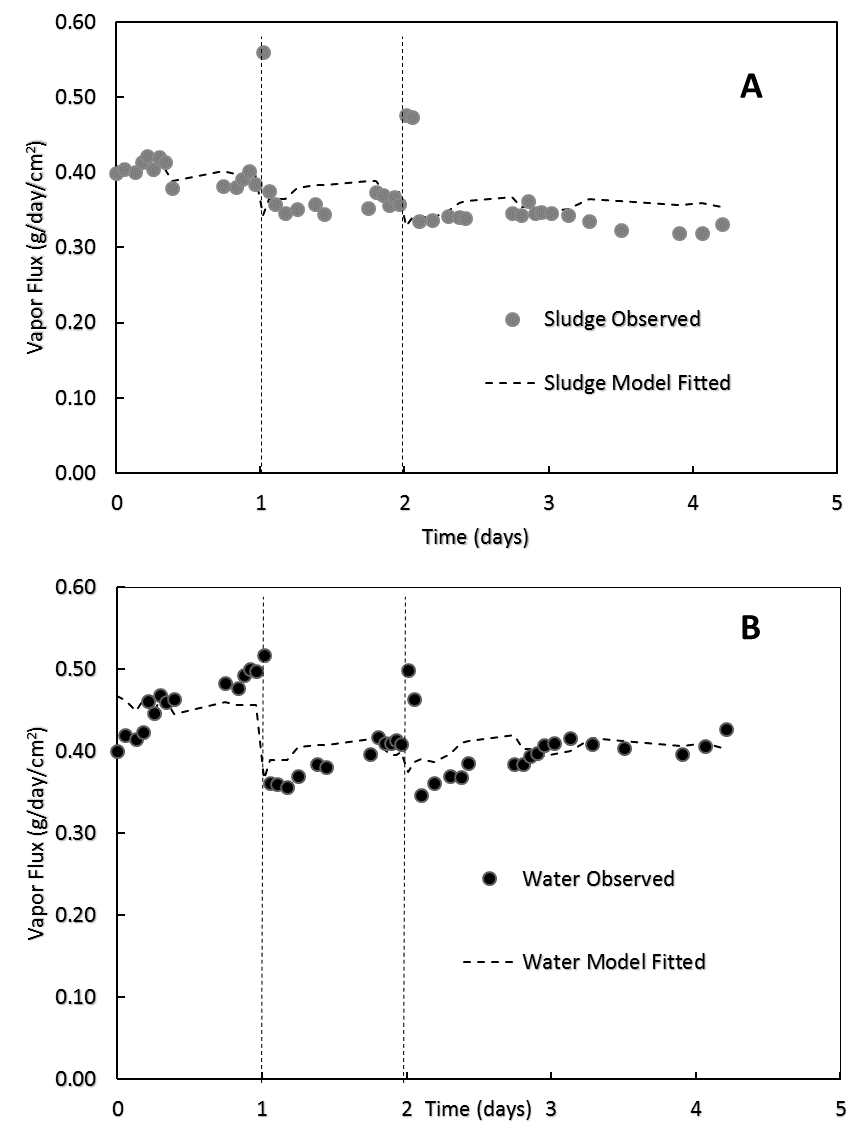


Figure S4. Observed and predicted drying rate per wetted surface area (WSA) (g/day/cm^2^) for A) fecal sludge and B) DI water (as control) using laminate boxes. The predicted drying rates are calculated using best-fit λ for Sludge1, Sludge2, Sludge3, Water1, Water2 and Water3 cycles, shown separated by vertical dotted lines. A capillary fringe was added to the water boxes’ WSA and a temperature correction of T_1_ = T_2_ used for Sludge1 and Water1 boxes.

1. **Full-Scale Membrane Drum Constant Temperature Room Test - Results**


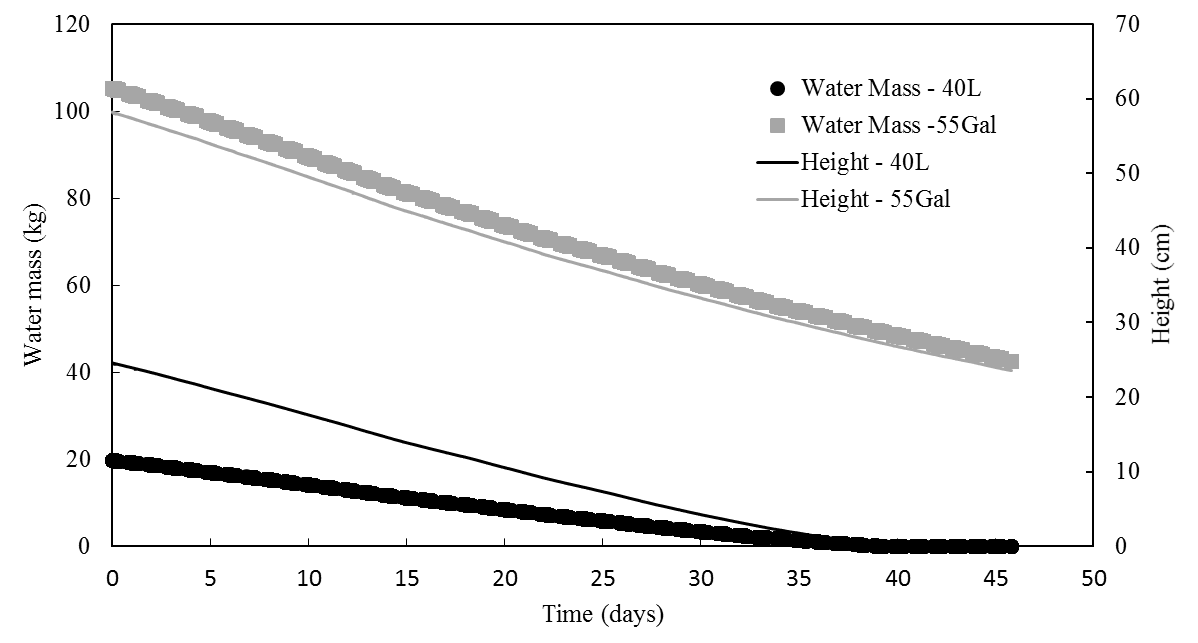


Figure S5. Water drying data for laminate-lined 40 L and 55-gallon drums in a constant temperature room with temperature 30°C and relative humidity 25%. Water mass and water depth are shown.


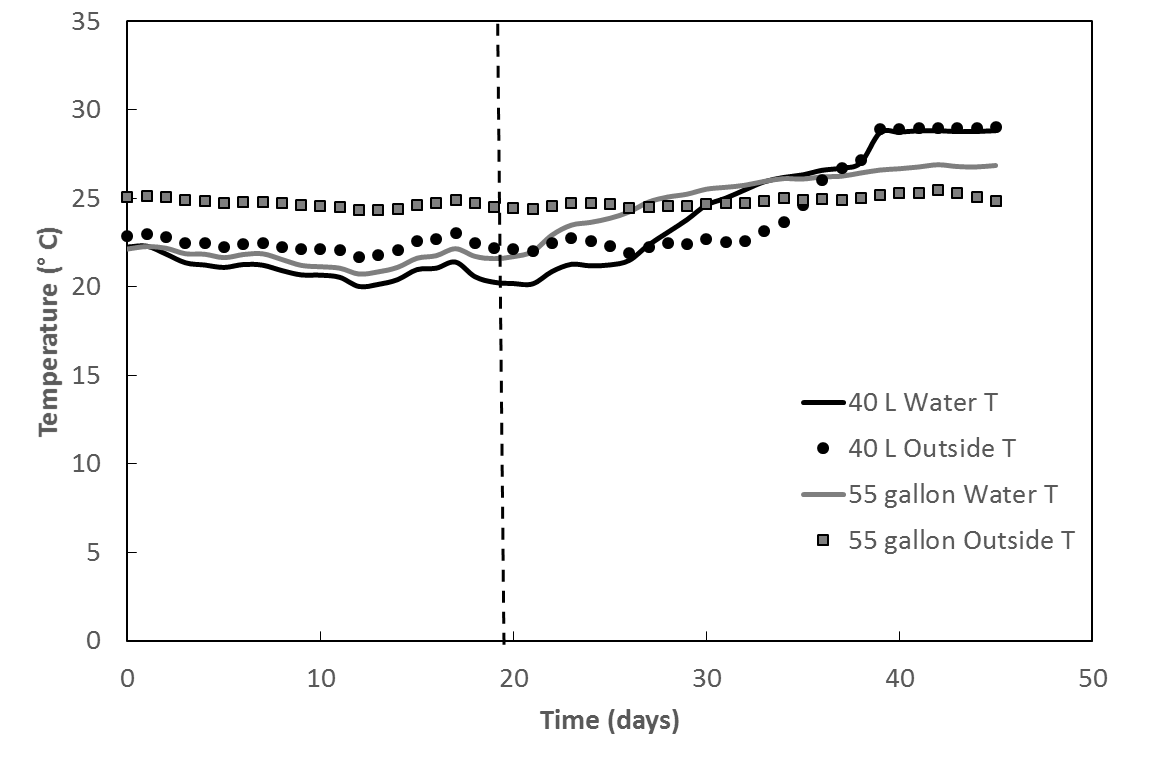


Figure S6. Water and outside air temperatures for laminate-lined 40 L and 55-gallon drums in a constant temperature room maintained at temperature 30°C and relative humidity 25%. Outside air temperatures were measured in the air space between the laminate and the plastic drum walls. Data after the vertical dashed line were not used for analysis since the thermocouple sensors were no longer submerged in water after $\approx2$0 days.

1. **Full-Scale Membrane Drum Outside Test - Results**


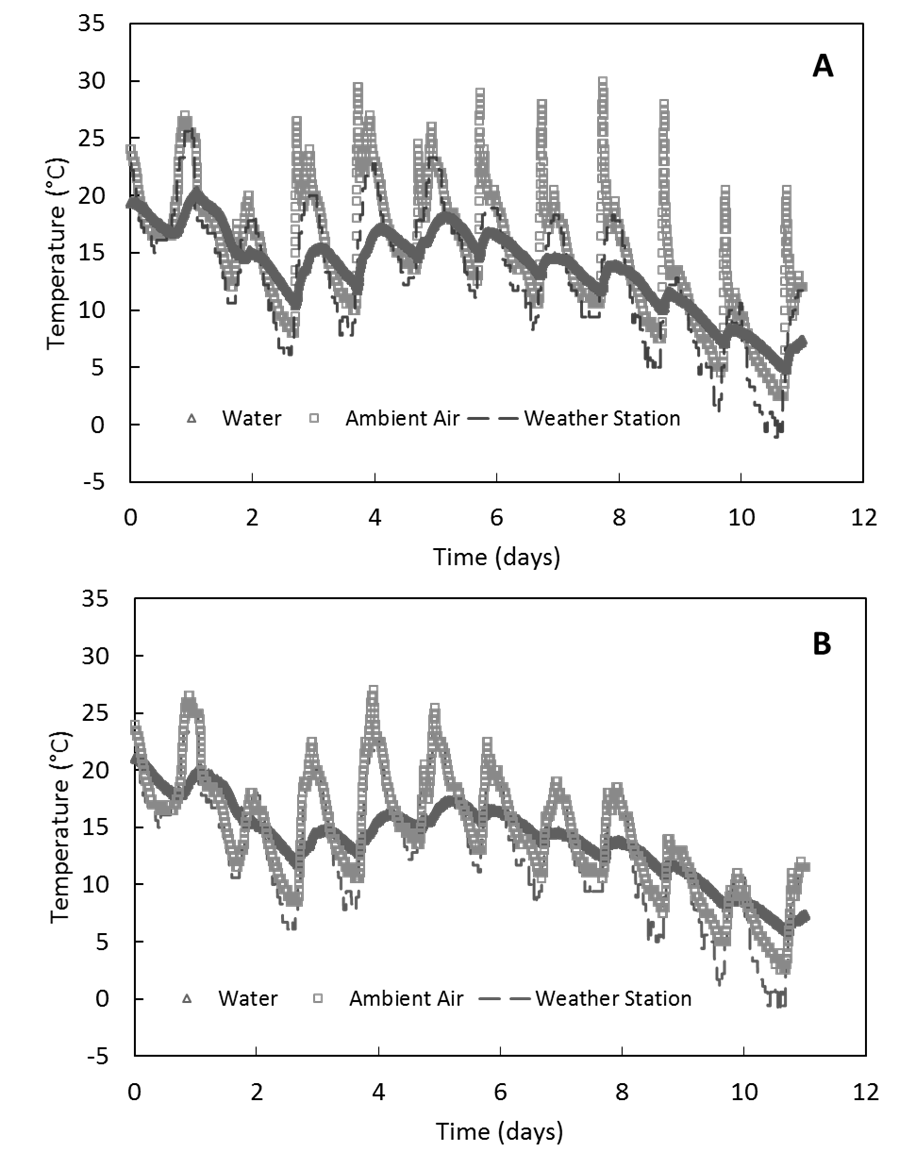


Figure S7. Bulk water temperature, ambient air temperature, and local climatological air temperature for the laminate-lined A) 40 L and B) 55-gallon drums. Tests conducted outside.


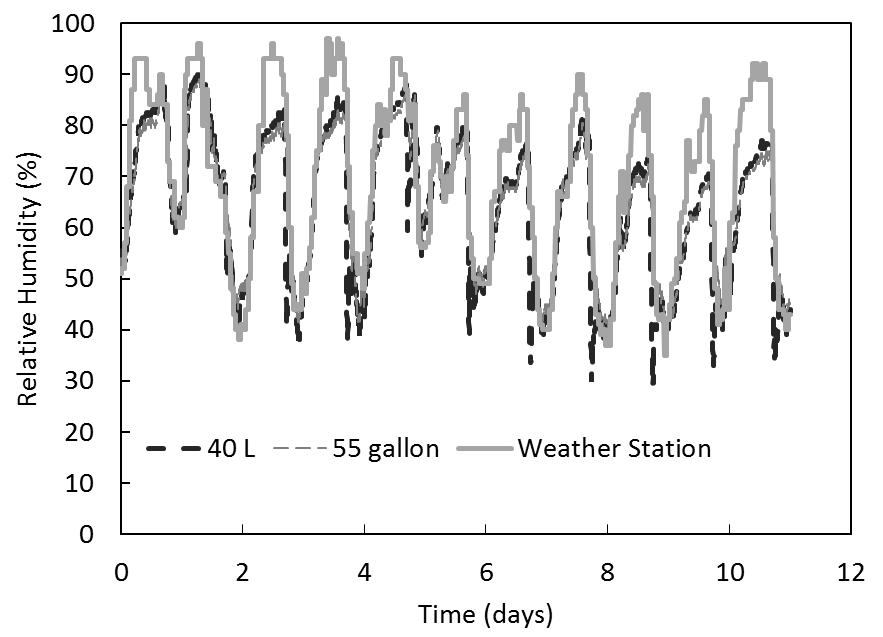


Figure S8. Relative humidity measured near the drums and the local climatological relative humidity for the laminate-lined 40 L and 55-gallon drums.

**References**

GIBSON, P. W. 2000. Effect of temperature on water vapor transport through polymer membrane laminates. *Polymer Testing,* 19**,** 673; 673.

KHAYET, M., VELÁZQUEZ, A. & MENGUAL, J. I. 2004. Direct contact membrane distillation of humic acid solutions. *MEMSCI</cja:jid> Journal of Membrane Science,* 240**,** 123-128.

MARZOOGHI, S., SHI, C., DENTEL, S. K. & IMHOFF, P. T. 2017. Modeling biosolids drying through a laminated hydrophobic  membrane. *Water Research***,** 244-253.
